# Effectiveness of Integrated Digital Solutions to Empower Older Adults in Aspects Related to Their Health: Systematic Review and Meta-Analysis

**DOI:** 10.2196/54466

**Published:** 2025-01-09

**Authors:** Ana Isabel Martins, Óscar Ribeiro, Gonçalo Santinha, Telmo Silva, Nelson P Rocha, Anabela G Silva

**Affiliations:** 1 Center for Health Technology and Services Research University of Aveiro Aveiro Portugal; 2 Department of Education and Psychology Center for Health Technology and Services Research University of Aveiro Aveiro Portugal; 3 Department of Social, Political and Territorial Sciences Research Unit in Governance, Competitiveness and Public Policies University of Aveiro Aveiro Portugal; 4 Department of Communication and Art DigiMedia University of Aveiro Aveiro Portugal; 5 Department of Medical Sciences Institute of Electronics and Informatics Engineering of Aveiro University of Aveiro Aveiro Portugal; 6 School of Health Sciences Center for Health Technology and Services Research University of Aveiro Aveiro Portugal

**Keywords:** empowerment, older adults, digital health, digital solutions, effectiveness, empowerment, health related, outcomes, systematic review, meta-analysis, synthesize, evaluate, apps, mHealth, mobile health

## Abstract

**Background:**

Digital solutions, such as mobile apps or telemonitoring devices, are frequently considered facilitators in the process of empowering older adults, but they can also act as a source of digital exclusion or disempowerment if they are not adequate for older adults’ needs and characteristics.

**Objective:**

This study aimed to synthesize and critically evaluate existing evidence on the effectiveness of integrated digital solutions that enable interaction for empowering older adults in aspects related to their health and to explore potential factors (eg, type of technology, participants’ characteristics) impacting effectiveness.

**Methods:**

A systematic search was carried out in PubMed, ScienceDirect, SCOPUS, EBSCO, and SciELO using a combination of terms informed by previous reviews on empowerment. Screening of references was performed against predefined inclusion criteria. Data extraction and the methodological quality of included studies using the PEDro (Physiotherapy Evidence Database) scale were performed by 2 authors. The certainty of evidence was graded for the main comparisons and outcomes of the review using the GRADE (Grading of Recommendations Assessment, Development, and Evaluation) framework. When at least 3 studies were available within the same domain of empowerment (knowledge, support by others, capacities, and behaviors) and comparison group, a meta-analysis was performed.

**Results:**

A total of 30 manuscripts were included in the review. Regarding knowledge, there was very low certainty of evidence of a medium effect size (ES) favoring the digital intervention group (k=5, ES=0.40, 95% CI 0.07-0.73, *I*^2^=79%). Regarding capacities, there was low certainty of evidence of no between-group differences (k=5, d=0.13, 95% CI –0.02 to 0.29, *I*^2^=0%) when comparing digital solutions against no intervention, low certainty of evidence of a medium ES favoring the digital intervention group (k=13, d=0.29, 95% CI 0.07-0.52, *I*^2^=79%) when comparing digital solutions against usual care, and very low certainty of evidence of no between-group differences (k=4, d=0.97, 95% CI –0.62 to 2.56, *I*^2^=97%) when comparing digital interventions to face-to-face interventions. Regarding social support and behaviors, no meta-analysis was possible, and existing evidence is conflicting.

**Conclusions:**

There is very-low-to-low certainty of evidence that using integrated digital solutions results in increased knowledge and increased capacities (mainly self-efficacy) when compared to usual care and impacts capacities to an extent similar to face-to-face interventions at postintervention. Interestingly, results also suggest, with low certainty of evidence, that there are no differences between using digital solutions and no intervention for improving capacities. Included studies and technologies were diverse, and meta-analysis showed high heterogeneity, which limits the confidence in the results and suggests that further research might affect the conclusions of this review.

**Trial Registration:**

PROSPERO CRD42022346823; https://tinyurl.com/39k29pzc

## Introduction

Empowerment is a complex concept with multiple definitions [1,2] and is affected by multiple factors, including individual and contextual factors [3]. One definition refers to empowerment as a social process of recognizing, promoting, and enhancing people’s ability to meet their own needs, solve their problems, and mobilize the necessary resources to feel in control of their own lives [4]. In other words, empowerment is a process by which people, organizations, and communities gain mastery over issues of concern to them [3]. When a person or group is empowered, they can make impactful decisions, allowing them to turn those decisions into the actions and results they desire [1,5].

Empowering older adults is of great importance as a means of promoting autonomy and independence by allowing them to make their own decisions, foster social connections that endorse social participation [1], and engage in activities that bring them joy and fulfillment [6]. In addition, empowering older adults contributes to ensuring that they are treated with dignity and respect; to acknowledging their life experiences, wisdom, and contributions to society [7]; and to fostering a sense of self-worth, value, and independence [8]. Empowered older adults feel encouraged to take control of their physical, mental, and emotional health and make informed decisions about their health care and well-being [8,9]. Overall, empowerment means having an opportunity to learn, discuss, decide, and act on decisions across all areas and dimensions of one’s own life [10].

Digital solutions, such as mobile apps or telemonitoring systems, are frequently considered facilitators in the process of empowering older adults [11] as they often provide access to a vast amount of information, resources, and services [12] and improve social connection and communication, particularly with family members and friends who do not live nearby [13]. This enables older adults to stay informed, engage in lifelong learning, have access to services and products, make informed decisions about their health and well-being [14], and maintain social connections regardless of physical distance. However, digital solutions can also act as a source of segregation, digital exclusion, or disempowerment for older people as they are not adequate for older adults’ needs and characteristics [15-17]. Interaction (ie, the ability to engage with a digital solution) is likely to be a key aspect of the potential of digital solutions for empowering as interactive solutions allow for a more personalized and immersive experience [18]. Nevertheless, several barriers hinder the effective use of digital solutions by older adults, and potentially its ability to empower them, such as the digital divide, the technological complexity, or the lack of flexibility and adequacy to their needs and characteristics, such as age-related or disease-related physical and cognitive constraints [19-21]. Therefore, the potential of digital solutions as a means of empowering older adults might not translate into an effective positive impact, or this impact might vary across different types of digital solutions or depend on the characteristics of older adults. However, and to the best of our knowledge, there is no available synthesis of evidence on the effectiveness of digital solutions for empowering older adults that clarifies the impact of digital solutions or that attempts to identify potential factors affecting their effectiveness. Thus, this study aimed to synthesize and critically evaluate existing evidence on the effectiveness of integrated digital solutions that enable interaction for empowering older adults in aspects related to their health and to explore potential factors (eg, type of technology, participants’ characteristics) impacting effectiveness.

## Methods

### Study Design

This study was registered in PROSPERO (International Prospective Register of Systematic Reviews; CRD42022346823). The PRISMA (Preferred Reporting Items for Systematic Reviews and Meta-Analyses) guidelines (Multimedia Appendix 1) were followed in this review [22].

### Eligibility Criteria

Studies were included in this review if they met the following inclusion criteria:

- Participants had a mean age of 60 years or more.
- Studies reported randomized or quasi-randomized trials aiming primarily at assessing the effectiveness of technology for empowering older adults.
- One of the arms used an integrated digital solution that enables interaction with the users. Examples include complex technologies, such as remote health and social monitoring solutions, and ambient assisted living solutions or simpler solutions, such as mobile phone apps and websites. A description and examples are presented in Multimedia Appendix 2.
- The comparison was no intervention, usual care, or any intervention (eg, education, written material) that did not use a digital solution.
- The outcome of interest was 1 of the following 4 dimensions of empowerment [23]: (1) patients’ capacities (tolerance of uncertainty, skill and technique acquisition, constructive attitudes and approaches, self-monitoring and insight, emotional well-being, positive attitude, self-efficacy, ownership, and self-information search), (2) patients’ knowledge (knowledge and confidence in decision-making, informed confidence), (3) patients’ behaviors (positive and active engagement in life, health-directed behavior, health service navigation, enabling others, social advocacy, commitment, informed choice, navigation, partnership, and health maintenance), and (4) support by others (social integration and support, integration, client-provider relationship, client-client support, control, doctors’ health locus of control, and other people’s health locus of control).
- Studies were published in English, Spanish, or Portuguese. Other languages were excluded because of limitations in the language abilities of the research team.

Studies with measurement instruments including individual items on empowerment but not providing individual item scores were excluded.

### Search Strategy

A systematic search was carried out in the following electronic databases: PubMed, ScienceDirect, SCOPUS, EBSCO, and SciELO. The search strategy consisted of a combination of terms based on previous reviews on empowerment [24] and technology [25] (for details, see Multimedia Appendix 3). In PubMed, the following search string was used: ((“Empowerment”[Mesh] OR “Patient Participation”[Mesh]) OR “self-efficacy” OR “locus of control” OR perceived autonomy OR perception of autonomy OR overconfidence) AND ((“Technology”[Mesh]) OR “Digital Technology”[Mesh] OR “Mobile apps” OR m-health OR ehealth OR e-health OR “digital solution” OR “Ambient Assisted Living” OR “digital services” OR website OR “digital platform” OR esocial). This string was adapted for the remaining databases. No filters were used. The search was conducted on May 4, 2022, and updated on September 6, 2022, and included papers from each database’s inception until August 31, 2023.

### Selection Process

After importing the papers into Mendeley (Elsevier), duplicates were removed. Next, the papers were imported to CADIMA (Julius Kühn-Institut), the software used to support screening, which was performed first by title and abstract and then by full text against the eligibility criteria. The entire selection process was performed independently by 2 authors (AIM and AGS). Disagreements or discrepancies during the selection process were resolved by consensus or consultation with a third author (NPR or OR).

### Data Extraction and Management

A customized Microsoft Excel form was used for data extraction, which was piloted using 3 studies. The data extraction was manually completed by 1 author (AIM) and checked by a second author (AGS). The following data were extracted from each study: author and date, measurement instruments used to assess the outcomes of interest, intervention (type and duration), characteristics of the sample per group (health condition, sample size, age, sex), comparison, outcomes, and results (at postintervention and follow-up assessments). Data on the period of follow-up and the results of follow-up assessments were collected to allow for analysis of the effectiveness of digital solutions at different time points. When multiple outcomes within the same domain of empowerment were reported in the same paper, the choice of which to include in the meta-analysis was based on similarity with outcomes from other studies in the same meta-analysis. When data were not fully accessible (eg, presented in graphs), they were not extracted and authors were contacted.

Extracted data were summarized under 1 of the 4 domains of empowerment previously defined (patients’ capacities, patients’ behaviors, patients’ knowledge, and support by others). When necessary, the transformation of data, such as the conversion of SEs and CIs into SDs, was performed according to Cochrane recommendations [26].

### Methodological Quality Assessment

The quality of studies was assessed using the PEDro (Physiotherapy Evidence Database) scale, which is a valid instrument [27]. Each study was assessed independently by pairs of reviewers (AIM and AGS, NPR and OR), and disagreements were resolved by a third author. The PEDro scale is composed of 11 items, and each item is scored as either present (1) or absent (0). The first item is not considered in the total score, leading to a maximum score of 10. The total score can be interpreted as follows: 0-3, poor; 4-5, fair; 6-8, good; and 9-10, excellent methodological quality [28].

### Assessment of the Certainty of Evidence

The certainty of evidence was graded for the main comparisons and outcomes of this review. The overall quality and strength of the evidence per outcome was assessed according to the GRADE (Grading of Recommendations Assessment, Development, and Evaluation) framework. Evidence was assessed against (1) risk of bias, (2) inconsistency of results, (3) indirectness, (4) imprecision, and (5) publication bias, and the overall quality of evidence was classified as high, moderate, low, or very low [29,30]. We started with high evidence as studies included in the meta-analysis were randomized trials, and then, the evidence was downgraded, considering the researchers’ assessment of each criterion.

### Data Synthesis

When at least 3 studies were available within the same empowerment domain and comparison group (no intervention, usual care, or face-to-face intervention), a meta-analysis was performed. In addition, separate analyses were performed considering whether the digital intervention was the only intervention or was administered together with a face-to-face intervention. A sensitivity analysis was performed excluding lower-quality studies. Effect sizes (ESs) and their 95% CIs were determined by the standardized mean difference (SMD) and classified according to Cohen’s guidelines as small (0.20), medium (0.50), and large (0.80) effects [31]. Statistical heterogeneity was investigated using *I*^2^ statistics interpreted as 25%, 50%, and 75%, reflecting low, moderate, and high statistical heterogeneity, respectively. When at least 10 studies were available, funnel plots were generated to inform publication bias. Follow-up periods were chosen to increase the similarity between studies in the same meta-analysis. The meta-analysis was performed using R version 4.2.2 (R Foundation for Statistical Computing). When no meta-analysis was possible, a qualitative synthesis was performed, indicating the comparisons investigated by the studies and the direction of the effect reported.

## Results

### Study Details

**Figure 1:**
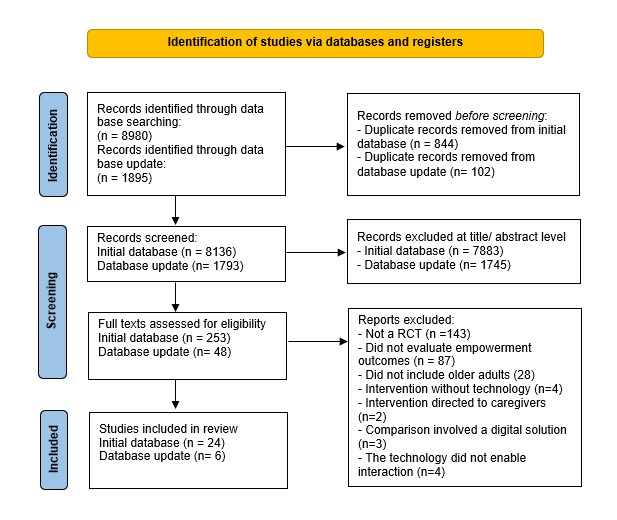
Flow diagram of the current review.

A total of 10,875 references were found, of which 946 (8.7%) were duplicates. The remaining 9929 (91.3%) references were screened by title and abstract, and 301 (3%) full-text manuscripts were retrieved and read. Of these, 30 (10%) manuscripts were included in the review (Figure 1). The included manuscripts assessed the impact of using a variety of digital solutions, including the following: websites (n=6, 20%), general mobile apps (n=4, 13.3%), condition-specific mobile apps (n=6, 20%), desktop software (n=1, 3.3%), remote monitoring (n=6, 20%), remote monitoring plus a website (n=1, 3.3%), remote monitoring plus mobile apps (n=5, 16.7%), and remote monitoring, a specific mobile app, plus a website (n=1, 3.3%).

### Quality and Certainty of Evidence

The studies scored between 3 and 8 on the PEDro scale. Most studies scored 6-8 (n=20, 66.7%), suggesting good quality; 9 (30%) studies scored between 4 and 5, suggesting fair quality; and 1 (3.3%) study scored 3, suggesting poor quality (Multimedia Appendix 3). The certainty of evidence varied between very low to low and is presented next, along with the results of each meta-analysis (Multimedia Appendix 4).

### Impact of Digital Interventions on Empowerment at Postintervention

Data extracted from individual studies are presented in Multimedia Appendix 5.

#### Patients’ Behaviors

Four studies [32-35] assessed the effect of digital solutions on behavior. One study [34] with fair quality compared a digital solution to usual care for patients with chronic obstructive pulmonary disease (COPD) and reported no between-group differences in self-management behavior. Two studies (one with good and the other with fair methodological quality) found the group receiving the digital intervention to report increased self-care behaviors when compared to usual care [32] and to a large written poster [35]. The other study [33], with fair quality, reported no differences between the group receiving the digital intervention and the no-intervention group for perceived participation in enjoyable activities. No meta-analysis was possible due to the unavailability of needed data.

#### Support by Others

Six studies [35-40] investigated the impact of digital interventions on support by others. Of these, 2 (33.3%) studies (with fair and good methodological quality) compared digital interventions against no intervention: one reported no between-group differences [39], while the other reported that the digital intervention significantly increased social support compared to no intervention [38]. Another study [37], with fair quality, reported no differences in social support between the group receiving the digital intervention and the group receiving usual care. Two studies (fair and good quality) [35,36] used a digital intervention plus a face-to-face component: one reported no significant differences compared to no intervention [36], while the other reported a significant improvement in social support compared to a written large poster [35]. Another study [40] of good quality reported a significant difference at 12 months between the digital intervention and a paper-based health education program, favoring the latter (medium ES).

#### Patients’ Knowledge

**Figure 2:**
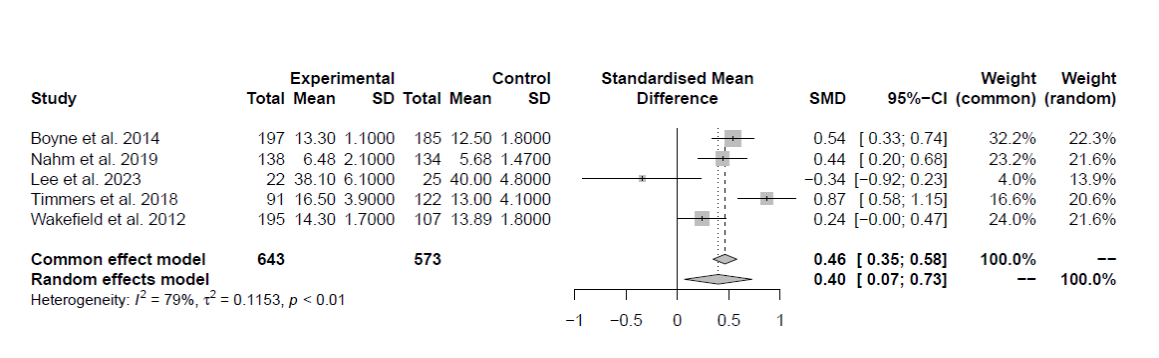
Plot of the meta-analysis of the standardized mean differences for patients’ knowledge (comparison: digital solutions versus usual care or education).

Seven studies [32,37,41-45] investigated the impact of digital technologies on patients’ knowledge. Of these, 3 (42.9%) studies [32,44,45], 2 (66.7%) of good quality and 1 (33.3%) of fair quality, compared digital solutions against usual care; another 2 (28.6%) studies [41,42], both of fair quality, compared digital solutions against education. There was very low certainty of evidence of a medium ES favoring the experimental group (ES=0.40, 95% CI 0.07-0.73, *I*^2^=79%; Figure 2). The remaining 2 (28.6%) studies [37,43], not included in the meta-analysis, were of fair quality and reported digital solutions to have a greater impact on knowledge compared to usual care.

#### Patients’ Capacities

**Figure 3:**
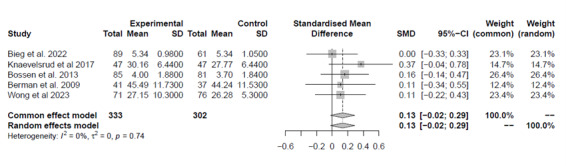
Plot of the meta-analysis of the standardized mean differences for patients’ capacities (comparison: digital solutions versus no intervention).

**Figure 4:**
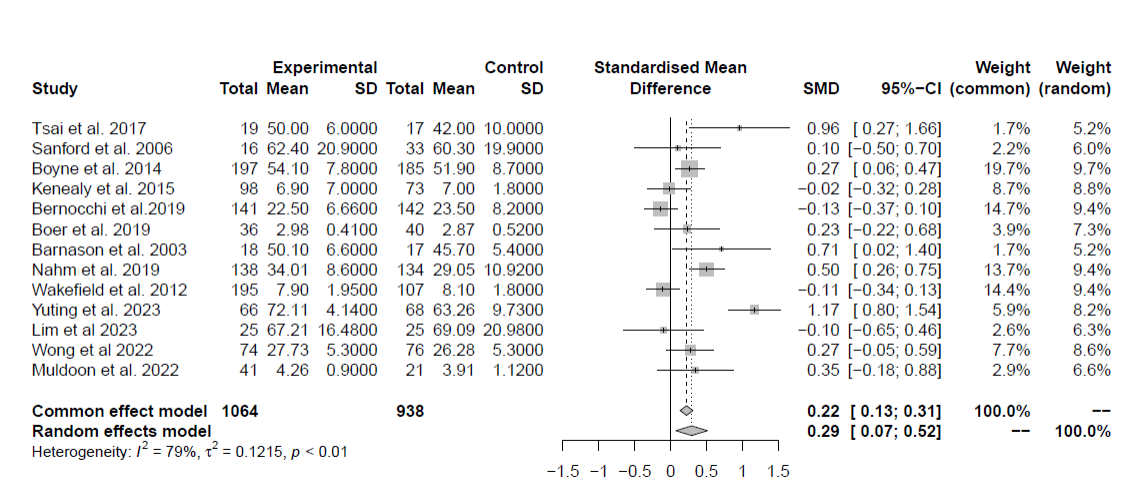
Plot of the meta-analysis of the standardized mean differences for patients’ capacities (comparison: digital solutions versus usual care).

Twenty-five studies investigated the impact of technology on older adults’ capacities. A meta-analysis of 5 (20%) studies (n=1, 20%, study with poor, n=1, 20%, study with fair, and 3, 60%, studies with good quality) [39,46-49] that compared digital solutions against no intervention found, with low certainty of evidence, no between-group differences (d=0.13, 95% CI –0.02 to 0.29, *I*^2^=0%; Figure 3). A meta-analysis of 13 (52%) studies (n=10, 76.9%, studies with good and n=3, 23.1%, studies with fair quality) [32,34,40,44,45,50-57] comparing digital solutions against usual care indicated, with low certainty of evidence, a medium ES favoring the digital intervention group (d=0.29, 95% CI 0.07-0.52, *I*^2^=79%; Figure 4). Sensitivity analysis including the 10 (76.9%) studies with good quality did not affect the results (d=0.27, 95% CI 0.02-0.53, *I*^2^=81%).

**Figure 5:**
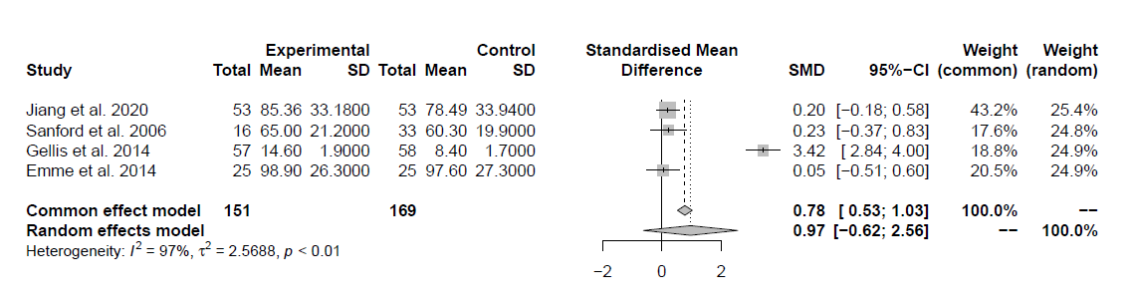
Plot of the meta-analysis of the standardized mean differences for patients’ capacities (comparison: digital interventions to face-to-face interventions).

A meta-analysis of 4 (16%) studies [51,58-60] with good quality that compared digital interventions to face-to-face interventions suggested, with very low certainty of evidence, no between-group differences (d=0.97, 95% CI –0.62 to 2.56, *I*^2^=97%; Figure 5).

Of the 4 (16%) studies [35-37,61] not included in any of the meta-analyses, 3 (75%) of fair-to-good quality reported no between-group differences when the digital solution was compared against usual care/phone calls [37,61] or combined with a face-to-face component and compared against no intervention [36]. The fourth study [35] presented fair quality and reported a significant difference favoring the group that received a digital solution when compared to face-to-face education.

### Impact of Digital Interventions on Empowerment at Follow-Up

#### Patients’ Behaviors

Only 1 (3.3%) study [32] assessed the effect of technology on behavior at follow-up (6 and 12 months) and reported that the group receiving the digital intervention reported increased self-care behaviors when compared to usual care.

#### Support by Others

Two studies [38,39] investigated the impact of digital interventions against no intervention on support by others at up to 12 months’ follow-up and reported no between-group differences.

#### Patients’ Knowledge

Three studies investigated the impact of digital interventions against usual care on patients’ knowledge at 3 [43], 4 [45], and up to 12 [32] months’ follow-up. Two studies [32,43] reported a significant difference in favor of the experimental group, while the third study [45] reported no between-group differences.

#### Patients’ Capacities

Two studies investigated the effect of digital solutions at 12 months’ follow-up compared to no intervention: one reported no between-group differences [39], while the other reported significant improvements in self-efficacy in the intervention group [48]. Two studies investigated the effect of digital solutions against face-to-face intervention and reported no between-group differences at 3 [59] and 6 [58] months’ follow-up. Three studies investigated the impact of digital solutions against usual care: 2 (66.7%) reported no between-group differences at 4 [45] and 12 [32] months’ follow-up, but 1 (33.3%) [53] reported increased self-efficacy in the digital solution group at 3 months’ follow-up.

## Discussion

### Principal Findings

This review synthesized existing evidence on the effectiveness of using integrated digital solutions for empowering older adults. Results suggest with very-low-to-low certainty of evidence that using digital solutions results in increased knowledge and increased capacities when compared to usual care and impacts capacities to an extent similar to face-to-face interventions. Interestingly, results also suggest, with low certainty of evidence, that there are no differences between using digital solutions and no intervention for improving capacities.

### Comparison With Prior Work

Our results show an apparent contradiction as they suggest that digital solutions are better than usual care and equal to face-to-face interventions at improving patients’ capacities but equal to no intervention. This might be explained by the characteristics of usual care, which, in the studies included, seems to equate to minimal care delivered over 1 or few sessions. As previously reported [62,63], usual care is generally not described in sufficient detail, hindering the interpretation of results. In addition, differences in the degree of interaction and support provided by the digital solutions, in the training given to individuals before the trial, in the amount of interaction with health professionals using the digital solutions, and in the personal and clinical characteristics of the participants might contribute to the differences found. Furthermore, the very-low-to-low certainty of the evidence, the wide CIs for the estimate of the effect, and the high heterogeneity in the usual-care meta-analyses suggest that further research is likely to impact the conclusions of the comparison of digital interventions against no intervention and usual care. Worth highlighting are the 2 studies that compared digital solutions against face-to-face interventions at follow-up and that confirmed the postintervention results by showing no between-group differences. Most studies within the capacity domain assessed self-efficacy (ie, people’s beliefs in their own capabilities), which is reduced in older adults receiving care [64] and is an important determinant of health-related behavior change [65].

Digital interventions seem to improve older adults’ knowledge to a higher extent than usual care, which, as previously reported, was not detailed in the included studies. The instruments used to assess acquired knowledge are diverse, but most relate to knowledge regarding a specific clinical condition. Other systematic reviews have reported that digital solutions can improve one’s knowledge, for example, related to diabetes [66], and are better than usual care for eHealth literacy [67]. A previous study also reported that using digital solutions for seeking health-related information (eg, on specific diseases, medication, symptoms, and health promotion) is common among older adults [21], potentially facilitating adherence to digital interventions that promote knowledge acquisition.

This systematic review with meta-analysis results carefully suggests that the choice of using a digital solution or not might be left to individual preferences and that technology can be used to reach more individuals who would not have access to face-to-face empowerment strategies. Other studies have identified patients’ needs and preferences as important aspects to consider when deciding between in-person health care delivery or remote health care delivery [68,69]. In addition, the decision on whether to use a digital solution might depend on the domain of empowerment being specifically targeted as relevant for a particular individual, potentially choosing digital solutions to target knowledge and capacities and face-to-face interventions for other domains. Choosing a mixed approach where some domains of empowerment are targeted with face-to-face interventions, while others are targeted with digital interventions might also be an option. In addition, issues of security and privacy should be considered when choosing digital solutions for older adults, as they often have lower levels of digital literacy and consequently are more exposed to privacy violations, frauds, scams, and phishing attacks [70,71]. Developing digital solutions that have user-friendly interfaces and implementing privacy by design (ie, incorporating privacy features into the design of digital solutions, such as data minimization principles) are essential. Additionally, education and awareness are a means to minimize the risk and to empower older adults with the knowledge and skills to identify online risks [70].

The similarity of results found in this review for the domains of patients’ knowledge and capacities might suggest that increased knowledge might promote increased capacities by providing older adults with more knowledge about their health, resources, and management strategies. The study of causality was beyond the scope of this review. This result, rather, identifies a potential topic of study for future studies.

Regarding patients’ behaviors and support by others, it was not possible to aggregate the evidence in a meta-analysis due to insufficient studies with a similar comparison. The qualitative analysis of the studies included in our systematic review identified conflicting results regarding the impact of digital interventions. The factors previously identified as potential sources of heterogeneity might also contribute to the conflicting results.

Future studies should detail the intervention administered to the control group, particularly if it is usual care, as well as detail the functionalities of the digital solution provided, including training, interaction with professionals, and adherence to the intervention, as these factors might have an impact on the effectiveness of the digital intervention to facilitate empowerment. Future systematic reviews and meta-analyses should attempt to identify sources of heterogeneity that might impact the results, including the type of digital solution, the amount of support given to participants, participants’ characteristics (age, education, digital literacy), the length of the intervention, and subdomains within each domain of empowerment.

### Limitations

The studies included are highly diverse in terms of the type of digital solutions, health conditions, participants’ characteristics, and instruments used to assess empowerment. All these aspects are reflected in the high heterogeneity found and undermine the confidence in the results of the meta-analysis. We aimed to run a subgroup meta-analysis considering the type of technology and users’ characteristics, but the small number of studies precluded this. For some comparisons, a meta-analysis was not possible due to the reduced number of studies with a similar comparison. This might suggest that empowerment is more researched when related to health domains. Studies that were not based on empowerment theory were not included.

### Conclusion

There is very-low-to-low certainty of evidence that using integrated digital solutions results in increased knowledge and increased capacities (mainly self-efficacy) when compared to usual care and that it impacts capacities to an extent similar to face-to-face interventions at postintervention. Interestingly, results also suggest, with low certainty of evidence, that there are no differences between using digital solutions and no intervention for improving capacities. Included studies and technologies are diverse, and the meta-analysis showed high heterogeneity, which limits the confidence in the results and suggests that further research might affect the conclusions of this review. The limited number of studies precluded a subgroup analysis considering the type of technology and user characteristics.

## Appendix

Multimedia Appendix 1 PRISMA (Preferred Reporting Items for Systematic Reviews and Meta-Analyses) checklist.

Multimedia Appendix 2 Description and examples of integrated digital solutions.

Multimedia Appendix 3 Search strategy with keywords.

Multimedia Appendix 4 Grading of Recommendations Assessment, Development, and Evaluation (GRADE) summary.

Multimedia Appendix 5 Methodological quality assessment and extracted data from individual studies.

## Abbreviations

ES: effect size
GRADE: Grading of Recommendations Assessment, Development, and Evaluation
PEDro: Physiotherapy Evidence Database
PRISMA: Preferred Reporting Items for Systematic Reviews and Meta-Analyses
