# Supplementary material for: Effectiveness of Integrated Digital Solutions to Empower Older Adults in Aspects Related to Their Health: Systematic Review and Meta-Analysis
Source: J Med Internet Res. 2025 Jan 9;27:e54466. doi: 10.2196/54466 (PMC11757982; doi:10.2196/54466)
Supplement: Multimedia Appendix 2 [file jmir_v27i1e54466_app2.docx]

| **Technology type** | **Description** | **Example** |
| --- | --- | --- |
| Remote health and/ or social monitoring solutions | Refers to technologies or services, that enable healthcare delivery or social monitoring from a distance, typically using digital communication tools and devices. These solutions facilitate access to medical care and support outside of traditional clinical settings, allowing patients to receive diagnosis, treatment, and ongoing management remotely [1]. | Home telehealth device (Viterion; Bayer/Panasonic), The telemonitoring device (Health Buddy®) [2]. |
| Ambient assisted living solutions | Refer to technologies or services, that are designed to support and enhance the quality of life for older adults and individuals with disabilities by leveraging ambient intelligence and smart technologies within their living environments. These solutions aim to promote independent living, safety, health monitoring, and social connectedness while enabling individuals to remain in their own homes and communities for as long as possible [3]. | A telehealth device consisting of a small in-home monitor connected to an agency central station [4]. |
| Mobile phone apps | Refers to software programs specifically designed to run on mobile devices such as smartphones and tablets. These apps serve various purposes and can provide users with a wide range of functionalities, including communication, education, health monitoring, and more [5]. | A smartphone app for oral health management [6]. |
| Websites | Refers to web pages, including multimedia content, typically identified with a common domain name and published on at least one web server. Websites are accessed via the Internet using a web browser, and they serve various purposes, such as providing information, offering products or services or facilitating communication within users [7]. | An Interactive website (ElderTree) designed to improve quality of life, social connection, and independence [8]. |

## **Multimedia Appendix 2**

Description and examples of integrated digital solutions.

## References

1. Khorakhun C, Bhatti SN. Remote health monitoring using online social media systems, *Proceedings of 2013 6th Joint IFIP Wireless and Mobile Networking Conference, WMNC 2013*, 2013, doi: 10.1109/WMNC.2013.6548953.
2. Boyne JJJ, Vrijhoef HJM, Spreeuwenberg M, De Weerd G, Kragten J, Gorgels APM, Effects of tailored telemonitoring on heart failure patients’ knowledge,  self-care, self-efficacy and adherence: a randomized controlled trial. *Eur J Cardiovasc Nurs* Jun 2014; 13(3): 243–252. doi: 10.1177/1474515113487464.
3. Döbereiner B. Ambient assisted living systems on the rise. Accessed Apr 16, 2024. [Online]. Available: https://healthcare-in-europe.com/en/news/ambient-assisted-living-systems-on-the-rise.html
4. Gellis ZD, Kenaley BL, Have TT,. Integrated telehealth care for chronic illness and depression in geriatric home  care patients: the Integrated Telehealth Education and Activation of Mood (I-TEAM) study. *J Am Geriatr Soc* May 2014; 62(5):889–895. doi: 10.1111/jgs.12776.
5. Zhao J, Freeman B, Li M. Can mobile phone apps influence people’s health behavior change? An evidence review. *J Med Internet Res* Nov 2016; 18(11):e5692. doi: 10.2196/jmir.5692.
6. Lee KH, Choi YY, Jung ES. Effectiveness of an oral health education programme using a mobile application for older adults: a randomised clinical trial. *Gerodontology* 2021; doi: 10.1111/ger.12616.
7. Brugger N. Website history and the website as an object of study. New Media Soc Feb 2009; 11(1–2):115–132. doi: 10.1177/1461444808099574.
8. Gustafson  Sr DH et al. Effect of an eHealth intervention on older adults’ quality of life and health-related outcomes: a randomized clinical trial. *J Gen Intern Med* 2022; 37(3):521–530. doi: 10.1007/s11606-021-06888-1.
