# Supplementary material for: Effectiveness of Integrated Digital Solutions to Empower Older Adults in Aspects Related to Their Health: Systematic Review and Meta-Analysis
Source: J Med Internet Res. 2025 Jan 9;27:e54466. doi: 10.2196/54466 (PMC11757982; doi:10.2196/54466)
Supplement: Multimedia Appendix 3 [file jmir_v27i1e54466_app3.docx]

## Multimedia Appendix 3

**Table S1.** Search strategy with keywords.

| PUBMED | (("Empowerment"[Mesh] OR "Patient Participation"[Mesh]) OR "self-efficacy" OR "locus of control" OR perceived autonomy OR perception of autonomy OR overconfidence) AND (("Technology"[Mesh]) OR "Digital Technology"[Mesh] OR "Mobile apps" OR health OR m-health OR ehealth OR e-health OR "digital solution" OR "Ambient Assisted Living" OR "digital services" OR website OR "digital platform" OR esocial) |
| --- | --- |
| Science Direct | ("Empowerment" OR "Patient Participation") AND ("Technology" OR “Mobile apps” OR mhealth OR ehealth OR “Ambient Assisted Living”) AND ("older adults")  (“Locus of control” OR "self-efficacy") AND ("Digital Technology" OR “Mobile apps” OR mhealth OR m-health OR “Ambient Assisted Living” OR “digital services”) AND ("older adults") |
| EBSCO | ("Empowerment" OR "Patient Participation" OR autonomy) AND ("digital Technology" OR “Mobile apps” OR mhealth OR "digital solution" OR “Ambient Assisted Living” OR “digital services”) AND ("older adults") |
| SCOPUS | (("Empowerment" OR "Patient participation" OR autonomy) AND ("digital Technology" OR "Mobile apps" OR mhealth OR "digital solution" OR "Ambient Assisted Living" OR "digital services" ) AND ("older adults")) |
| Scielo | ("Empowerment") AND ("Technology") AND ("older adults") |
