# Supplementary material for: Effectiveness of Integrated Digital Solutions to Empower Older Adults in Aspects Related to Their Health: Systematic Review and Meta-Analysis
Source: J Med Internet Res. 2025 Jan 9;27:e54466. doi: 10.2196/54466 (PMC11757982; doi:10.2196/54466)
Supplement: Multimedia Appendix 4 [file jmir_v27i1e54466_app4.docx]

## Multimedia Appendix 4

GRADE summary.

| Studies and design | Risk of Bias | Inconsistency | Indirectness | Imprecision | Publication Bias | Quality of the evidence |
| --- | --- | --- | --- | --- | --- | --- |
| Knowledge | | | | | | |
| 5 RCTs | Downgraded one level. Serious limitations: downgraded one level.  Methodological quality was graded as 5 and 6 on the PEDro scale | Downgraded one level. High heterogeneity (I^2^ =79%). | Downgraded one level. High variability in terms of sample characteristics, digital solutions, and measurement instruments. | Unclear. | Unclear. A funnel plot was not possible due to the small number of studies in the meta-analysis (n=5). Of the 5 studies included, 2 reported no significant differences between groups. | ⨁〇〇〇  Very Low |
| Capacities (digital solutions vs. no intervention) | | | | | | |
| 5 RCTs | Downgraded one level. Serious limitations: downgraded one level.  Methodological quality on the PEDro scale was graded between 3 and 6 for 4 studies. | Not relevant (I^2^=0%). | Downgraded one level. High variability in terms of sample characteristics, digital solutions, and measurement instruments. | Unclear. Relatively small CI but includes no effect and potential effect. (d=0.13; 95% CI=-0.02; 0.29). | Not relevant. All studies reported no significant differences between groups. | ⨁⨁〇〇  Low |
| Capacities (digital solutions vs. usual care) | | | | | | |
| 13 RCTs | No downgrade. 10 RCTs graded 6 to 8 on PEDro scale. | Downgraded one level. High heterogeneity (I^2^ =79%). | Downgraded one level. High variability in terms of sample characteristics, digital solutions, and measurement instruments. | Not relevant. High sample size. | Not relevant. A relatively symmetrical funnel plot. | ⨁⨁〇〇  Low |
| Capacities (digital solutions vs. face-to-face interventions) | | | | | | |
| 4 RCTs | No downgrade. All 4 RCTs graded 6 to 7 on PEDro scale. | Downgraded one level. High heterogeneity (I^2^=97%). | Downgraded one level. High variability in terms of sample characteristics, digital solutions, and measurement instruments. | Downgraded one level. Low sample size and high CI. | Not relevant. Three studies reported no significant differences between groups. | ⨁〇〇〇  Very Low |

Legend: RCT – randomized clinical trial; CI – confidence interval.
