# Supplementary material for: Effectiveness of Integrated Digital Solutions to Empower Older Adults in Aspects Related to Their Health: Systematic Review and Meta-Analysis
Source: J Med Internet Res. 2025 Jan 9;27:e54466. doi: 10.2196/54466 (PMC11757982; doi:10.2196/54466)
Supplement: Multimedia Appendix 5 [file jmir_v27i1e54466_app5.docx]

## Multimedia Appendix 5

**Table S2.** Methodological quality of included studies (1. Eligibility criteria; 2. Random allocation; 3. Concealment; 4. Groups similar at baseline; 5. Blinding of subjects; 6. Blinding of who administered the intervention; 7. Blinding of assessors; 8. Measures of outcome for 85% of subjects; 9. Treatment or control as allocated; 10. Statistics for between-group comparisons; 11. Point and variability measures.

| Authors (year) | 1 | 2 | 3 | 4 | 5 | 6 | 7 | 8 | 9 | 10 |  | Total (0-10)* |
| --- | --- | --- | --- | --- | --- | --- | --- | --- | --- | --- | --- | --- |
| Bieg et al (2022) [1] | 1 | 1 | 1 | 0 | 0 | 0 | 0 | 0 | 0 | 1 | 0 | 3 |
| Gustafson et al (2022) [2] | 1 | 1 | 1 | 1 | 0 | 0 | 0 | 1 | 1 | 1 | 1 | 7 |
| Lee et al (2023) [3] | 1 | 1 | 0 | 1 | 0 | 0 | 0 | 0 | 1 | 1 | 1 | 5 |
| Timmers et al (2018) [4] | 1 | 1 | 0 | 1 | 0 | 1 | 0 | 0 | 1 | 1 | 1 | 6 |
| Guo et al (2017) [5] | 1 | 1 | 0 | 1 | 0 | 0 | 0 | 1 | 1 | 1 | 0 | 5 |
| Tsai et al (2017) [6] | 1 | 1 | 1 | 1 | 0 | 0 | 1 | 1 | 1 | 1 | 1 | 8 |
| Jiang et al (2020) [7] | 1 | 1 | 0 | 1 | 0 | 0 | 0 | 1 | 1 | 1 | 1 | 6 |
| Wong et al (2021) [8] | 1 | 1 | 1 | 1 | 0 | 0 | 1 | 1 | 1 | 1 | 1 | 8 |
| Jung and Lee (2017) [9] | 1 | 0 | 0 | 1 | 0 | 0 | 0 | 1 | 1 | 0 | 1 | 4 |
| Knaevelsrud et al (2017) [10] | 1 | 1 | 0 | 1 | 0 | 0 | 0 | 1 | 1 | 1 | 1 | 6 |
| Wakefield et al (2012) [11] | 1 | 1 | 0 | 1 | 0 | 0 | 0 | 1 | 1 | 1 | 1 | 6 |
| Sanford et al (2006) [12] | 1 | 1 | 1 | 1 | 0 | 0 | 0 | 0 | 1 | 1 | 1 | 6 |
| Emme et al (2014) [13] | 1 | 1 | 1 | 1 | 0 | 0 | 0 | 1 | 1 | 1 | 1 | 7 |
| Li et al (2019) [14] | 1 | 1 | 0 | 1 | 0 | 0 | 0 | 0 | 0 | 1 | 1 | 4 |
| Boyne et al (2014) [15] | 1 | 1 | 0 | 1 | 0 | 0 | 0 | 1 | 1 | 1 | 1 | 6 |
| Kenealy et al (2015) [16] | 1 | 1 | 1 | 1 | 0 | 0 | 1 | 1 | 1 | 1 | 1 | 8 |
| Gellis et al (2014) [17] | 1 | 1 | 0 | 1 | 1 | 0 | 1 | 0 | 1 | 1 | 1 | 7 |
| Barnason et al (2003) [18] | 0 | 1 | 0 | 0 | 0 | 0 | 0 | 1 | 1 | 1 | 1 | 5 |
| Nahm et al (2019) [19] | 1 | 1 | 0 | 1 | 0 | 0 | 0 | 0 | 1 | 1 | 1 | 5 |
| Boer et al (2019) [20] | 1 | 1 | 0 | 0 | 0 | 0 | 0 | 1 | 1 | 1 | 1 | 5 |
| Fanning et al (2022) [21] | 1 | 1 | 0 | 1 | 0 | 0 | 0 | 1 | 1 | 1 | 1 | 6 |
| Bossen et al (2013) [22] | 1 | 1 | 1 | 1 | 0 | 0 | 0 | 0 | 1 | 1 | 1 | 6 |
| Bernocchi et al (2019) [23] | 1 | 1 | 1 | 1 | 0 | 0 | 0 | 1 | 1 | 1 | 1 | 7 |
| Berman et al (2009) [24] | 1 | 1 | 0 | 1 | 0 | 0 | 0 | 0 | 0 | 1 | 1 | 4 |
| Simbrig et al (2022) [25] | 1 | 1 | 1 | 1 | 0 | 0 | 0 | 0 | 1 | 1 | 0 | 5 |
| Wong et al (2023) [26] | 1 | 1 | 1 | 1 | 0 | 0 | 1 | 1 | 1 | 1 | 1 | 8 |
| Yuting et al (2023) [27] | 1 | 1 | 0 | 1 | 0 | 0 | 1 | 1 | 1 | 1 | 1 | 7 |
| Lim et al (2023) [28] | 1 | 1 | 1 | 0 | 0 | 0 | 1 | 1 | 1 | 1 | 1 | 7 |
| Wong et al (2022) [29] | 1 | 1 | 1 | 1 | 0 | 0 | 1 | 1 | 1 | 1 | 1 | 8 |
| Muldoon et al (2022) [30] | 1 | 1 | 1 | 0 | 0 | 0 | 1 | 1 | 1 | 1 | 1 | 7 |

Legend: 0 – No; 1 – Yes: * first item of the scale not included in the total score.

**Table S3.** Data extracted from studies assessing patients' capacities.

| Study and year | Instruments used to assess empowerment | Intervention (Digital solution group) | Comparison (control/no digital solution group) | Duration of intervention | Participants characteristics | | | | | | | Mean (SD) at post-intervention | |
| --- | --- | --- | --- | --- | --- | --- | --- | --- | --- | --- | --- | --- | --- |
|  |  |  |  |  | Health condition | Digital solution group | | | Control/no digital solution group | | | Digital solution group | Control/no digital solution group |
|  |  |  |  |  |  | Age, mean (SD) | n | Females, n (%) | Age, mean (SD) | n | Females, n(%) |  |  |
| Bieg et al (2022) [1] | BMPN subscale competence | Mobile app + wearable | No intervention | 12 months | No specific condition | 72.78 (6.48) | 89 | 64 (71.9) | 72.01 (6.59) | 61 | 47 (77.05) | 5.34 (0.98) | 5.34 (1.05) |
| Knaevelsrud, et al (2017) [10] | General Self-Efficacy Scale (GSE) | Website | No intervention | 6 weeks | Posttraumatic stress symptoms | 70.89 (4.97) | 47 | 35 (74.5) | 71.91 (4.48) | 47 | 26 (55.3) | 30.16 (6.44) | 27.77 (6.44) |
| Bossen, et al (2013) [22] | Arthritis Self-Efficacy Scale | Website | No intervention | 9 weeks | Knee and /or Hip Osteoarthritis | 61 (5.9) | 100 | 60 (60.0) | 63 (5.4) | 99 | 69 (69.7) | 4.0 (1.88) | 3.70 (1.84) |
| Berman et al (2009) [24] | Pain Self-efficacy Questionnaire (PSEQ) | Website | No intervention | 6 weeks | Chronic Pain | 64.3 (range (55-80) | 41 | 36 (87.9) | 67.5 range (57-91) | 37 | 32 (86.5) | 45.49 (11.73) | 44.24 (11.53) |
| Tsai et al (2017) [6] | Pulmonary Rehabilitation Adapted Index of Self-Efficacy (PRAISE) tool | Desktop software | Usual Care | 8 weeks | Chronic obstructive pulmonary disease (COPD) | 73 (8) | 19 | 7 (36.8) | 75 (9) | 17 | 11 (64.7) | 50 (6) | 42 (10) |
| Sanford et al (2006) [12] | Falls Efficacy Scale (FES) | Mobile app - General | Usual Care | 6 weeks | No specific condition | 57.8 (11.7) | 16 | 3 (16.1) | 62.7 (16.3) | 33 | 5 (16.10) | 62.4 (20.9) | 60.3 (19.9) |
| Boyne et al (2014) [15] | Barnason Efficacy Expectation Scale | Remote monitoring | Usual Care | Not clear | Heart failure | 71.0 (11.9) | 197 | 82 (41.6) | 71.9 (10.5) | 185 | 74 (40) | 54.1 (7.8) | 51.9 (8.7) |
| Kenealy et al (2015) [16] | Self-Efficacy for Managing Chronic Disease Scale | Remote monitoring | Usual Care | 3 to 6 months | Congestive heart failure, chronic obstructive pulmonary disease, diabetes, etc. | 65.3 (range 53-83) | 98 | 43 (43.8) | 69.75 (range 60-77) | 73 | 23 (31.5) | 6.9 (1.9) | 7.0 (1.8) |
| Bernocchi et al (2019) [23] | Falls Efficacy Scale  (FES) | Remote monitoring | Usual Care | 6 months | One or more chronic diseases (cardiac, respiratory, neuromuscular, or neurologic) | 77.9 (6.0) | 141 | 84 (60) | 79.3 (7.0) | 142 | 84 (59) | 22.5 (6.7) | 23.5 (8.2) |
| Wakefield et al (2011) [11] | Self-Efficacy to Manage Disease on General scale | Remote monitoring | Usual care | 6 weeks | Type 2 diabetes and hypertension | 68 (10)) | 195 | 4 (2) | 68 (10) | 107 | 2 (2) | 7.9 (2.0) | 8.1 (1.8) |
| Wong et al (2021) [8] | 10-item General Self-efficacy Scale | Mobile app - General | Phone Call | 3 months | Homebound during the COVID-19 pandemic | 72.2 (5.9) | 34 | 28 (82.4) | 71.3 (6.1) | 37 | 28 (82.4) | 30.3 (6.3) | 27.1 (5.7) |
| Li et al (2019) [14] | Hypertension Self-Efficacy Scale | Mobile app - General | Usual Care | 6 months | Hypertension | 61.7 (6.3) | 110 | 111 (59.7) | 61.3 (6.4) | 143 | 183 (65.9) | 0.8 (SD not provided) | -0.6 (SD not provided) |
| Jung and Lee (2017) [9] | 11-item scale to measure how confident an individual was at managing hypertension | Remote monitoring + Website+face-to-face intervention | Education | 24 weeks | Living alone with hypertension | 80.9 (6.6) | 31 | 24 (77.4) | 81.2 (4.1) | 33 | 29 (87.9) | 2.7 (0.5) | 2.1 (0.5) |
| Boer et al (2019) [20] | exacerbation-related self-efficacy scale | Remote monitoring + Mobile app - Specific | Usual Care | 12 months | No specific conditions | 69.3 (8.8) | 43 | 18 (41.9) | 65.9 (8.9) | 44 | 15 (34.1) | 2.98 (0.41) | 2.87 (0.52) |
| Barnason et al (2003) [18] | Barnason Efficacy Expectation Scale (BEES) | Remote monitoring | Usual Care | 6 weeks | Ischemic heart failure | 73.72 (5.06) | 18 | 4 (22) | 72.82 (4.80) | 17 | 7(41) | 50.1(6.6) | 45.7(5.4) |
| Jiang et al (2020) [7] | Exercise self-regulatory efficacy scale (Ex-SRES) | Mobile app - General | Face-to-face rehabilitation | 3 months | Chronic obstructive pulmonary disease | 70.92 (6.38) | 53 | 9 (17) | 71.83 (7.60) | 53 | 10 (19) | 85.4(33.2) | 78.5(33.9) |
| Nahm et al (2019) [19] | Four-item Self-Efficacy  for Computer-Based Personal Health Record (PHR) scale | Website | Usual Care | 3 weeks | Chronic Illnesses | 69.7 (8.6) | 138 | 93 (67.4) | 70.4 (8.5) | 134 | 98 (73.1) | 34.0 (8.6) | 29.1 (10.9) |
| Yuting et al (2023) [27] | Hypertension  Self-efficacy Scale | Remote monitoring + Mobile app – Specific | Usual care | 12 weeks | Hypertension | 61.37 (11.73) | 66 | 21 (31.82) | 62.09 (10.66) | 68 | 30 (44.12) | 72.11 (4.14) | 63.26 (26) |
| Lim et al (2023) [28] | Exercise self-efficacy scale (ESES) | Website | Paper-based health education program | 6 weeks | No specific condition | 73.5 (7.1) | 25 | 20 (60) | 73.6 (5.5) | 25 | 15 (80) | 67.21 (16.48) | 69.09 (20.98) |
| Wong et al (2022) [29] | General Self-efficacy Scale | Mobile app - Specific | Usual Care | 3 months | Chronic pain,  hypertension, or diabetes | 74.7 (7.6) | 74 | 60 (81.1) | 77.4 (8.2) | 76 | 63 (82.9) | 27.73 (5.3) | 26.28 (5.3) |
| Emme et al (2014) [13] | COPD self-efficacy scale (CSES) | Remote monitoring + Mobile app - General | Usual Care | Not clear (after discharge) | Chronic obstructive pulmonary disease | 71(FAZER SD a partir da Mediana) | 25 | 14 (56) | 74 | 25 | 15 (60) | 90.4 (22.8) | 97.6 (27.3) |
| Fanning et al (2022) [21] | 8-item scale wherein participants noted their confidence in their ability to walk over incrementally longer durations without stopping | Remote monitoring + Mobile app – Specific+face-to-face | No intervention | 12 weeks | Chronic pain | 70.12 (5.43) | 15 | 13 (86.7) | 70.32 ± 5.20 | 13 | 9 (69.2) | 59.8 (8.7) | 43.5 (8.3) |
| Wong et al (2023) [26] | Chinese version of the General Self-Efficacy Scale  (GSES) | Mobile app - Specific | No intervention | 12 weeks | Hypertension, diabetes, or chronic pain | 76.6 (8.0) (all) | 71 | 62 (87.3) | 76.6 (8.0) (all) | 76 | 63 (82.9) | 27.15 (10.3) | 26.28 (5.3) |
| Muldoon et al (2021) [30] | A six-item  scale for hypertension self-efficacy was developed for this study | Mobile app – Specific | Usual care | Unclear | Hypertension | 65.7 (range 55.0-86.0) | 41 | 29 (47) | 67.9 (range 55.0-83.0) | 21 | 11 (18) | 4.26 (0.90) | 3.91 (1.12) |
| Gellis et al (2014) [17] | Social Problem-Solving Inventory—Revised26 (SPSI-R) | Remote monitoring | Usual Care + education | 3 months | Medically frail older homebound individuals | 80.1 (7.8) | 57 | 36 (62.7) | 78.3 (6.9) | 58 | 40 (68.6) | 14.6 (1.9) | 8.4 (1.7) |

Legend: SD – Standard Deviation; n – sample size.

**Table S4.** Data extracted from studies assessing patients' knowledge.

| Study and year | Instruments used to assess empowerment | Intervention (Digital solution group) | Comparison (control/no digital solution group) | Duration of intervention | Participants characteristics | | | | | | | Mean (SD) at post-intervention | |
| --- | --- | --- | --- | --- | --- | --- | --- | --- | --- | --- | --- | --- | --- |
|  |  |  |  |  | Health condition | Digital solution group | | | Control/no digital solution group | | | Digital solution group | Control/no digital solution group |
|  |  |  |  |  |  | Age, mean (SD) | n | Females, n (%) | Age, mean (SD) | n | Females, n (%) |  |  |
| Boyne et al (2014) [15] | Dutch Heart Failure Knowledge Scale | Remote monitoring | Usual Care | Not clear | Heart failure | 71.0 (11.9) | 197 | 82 (41.6) | 71.9 (10.5) | 185 | 74 (40) | 13.3 (1.1) | 12.5 (1.8) |
| Nahm et al (2019) [19] | An eight-item questionnaire developed by experts and tested in other studies | Website | Usual Care | 3 weeks | Chronic Illnesses | 69.7 (8.6) | 138 | 93 (67.4) | 70.4 (8.5) | 134 | 98 (73.1) | 6.5 (1.2) | 5.68 (1.5) |
| Lee et al (2023) [3] | A self-report questionnaire | Mobile app - Specific | Education | 5 weeks | No specific condition | Data not provided | 22 | 18 (81.8) | Data not provided | 26 | 23 (88.5) | 38.1 (6.1) | 35.5 (7.8) |
| Wakefield et al (2011) [11] | 16-item multiple choice test focused on Diabetes and Hypertension | Remote monitoring | Usual care | 6 weeks | Type 2 diabetes and hypertension | 68 (10) | 195 | 4 (2) | 68 (10) | 107 | 2 (2) | 14.4 (1.7) | 13.9 (1.5) |
| Guo et al (2018) [5] | 11-item questionnaire on atrial fibrillation | Mobile app - Specific | Usual care | 1 month | Atrial fibrillation | 67.4 (10,6) | 91 | 46 (50) | 61.75 (8.54) | 122 | 66 (54.1) | Not reported | Not reported |
| Li et al (2019) [14] | 35-item questionnaire designed by Chinese guidelines for hypertensive patients’ education | Mobile app - General | Usual care | 6 months | Hypertension | 61.7 (6.3) | 110 | 111 (59.7) | 61.3 (6.4) | 145 | 183 (65.9) | 2.3 (7.9) | 0.8 (7.3) |
| Timmers et al (2018) [4] | Perceived knowledge questionnaire | Mobile app - Specific | Education | Not reported | Osteoarthritis | 62.27 (8.32) | 91 | 46 (50) | 61.75 (8.54) | 122 | 66 (54.1) | 16.5 (3.9) | 13.0 (4.1) |

Legend: SD – Standard Deviation; n – sample size.

**Table S5.** Data extracted from studies assessing “support by others”.

| Study and year | Instruments used to assess empowerment | Intervention (Digital solution group) | Comparison (control/no digital solution group) | Duration of intervention | Participants characteristics | | | | | | | Mean (SD) at post-intervention | |
| --- | --- | --- | --- | --- | --- | --- | --- | --- | --- | --- | --- | --- | --- |
|  |  |  |  |  | Health condition | Digital solution group | | | Control/no digital solution group | | | Digital solution group | Control/no digital solution group |
|  |  |  |  |  |  | Age, mean (SD) | n | Females, n (%) | Age, mean (SD) | n | Females, (F) n(%) |  |  |
| Bieg et al (2022) [1] | WHOQOL-OLD subscale of social participation consists of four items | Mobile app + wearable | No intervention | 12 months | No specific conditions | 72.78 (6.48) | 89 | 64 (71.9) | 72.01 (6.59) | 61 | 47 (77.05) | 75.5 (15.8) | 77.08 (13.97) |
| Gustafson et al (2022) [2] | Medical Outcomes Study (MOS) Social Support Survey | Website | No intervention | 12 months | No specific conditions | 76.3 (7.4) | 197 | 145 (73.6) | 76.8 (7.5) | 193 | 147 (76.2) | 3.7 (1.0) | 3.53 (1.0) |
| Li et al (2019) [14] | Social Support Rating Scale | Mobile app - General | Usual Care | 6 months | Hypertension | 61.7 (6.3) | 110 | 111 (59.7) | 61.3 (6.4) | 143 | 183 (65.9) | 0.4 (SD not provided) | 0.7 (SD not provided) |
| Jung and Lee (2017) [9] | Multidimensional Scale of Perceived Social Support | Remote monitoring + Website+ + face-to-face education | Education | 24 weeks | Living alone with hypertension | 80.9 (6.6) | 31 | 24 (77.4) | 81.2 (4.1) | 33 | 29 (87.9) | 55.9 (16.1) | 43.8 (17.4) |
| Lim et al (2023) [28] | HLQ scale 4, social support for health | Website | Paper-based health education program | 6 weeks | No specific condition | 73.5 (7.1) | 25 | 20 (60) | 73.6 (5.5) | 25 | 15 (80) | 3.12 (0.54) | 3.30 (0.61) |
| Fanning et al (2022) [21] | The 36-Item Short Form Survey (SF-36) to evaluate the social functioning domain | Remote monitoring + Mobile app – Specific+ + face-to-face education | No intervention | 12 weeks | Chronic pain | 70.12 (5.43) | 15 | 13 (86.7) | 70.32 (5.20) | 13 | 9 (69.2) | 64.3(8.5) | 69.5 (8.2) |

Legend: SD – Standard Deviation; n – sample

**Table S6.** Data extracted from studies assessing “ behaviours”.

| Study and year | Instruments used to assess empowerment | Intervention (Digital solution group) | Comparison (control/no digital solution group) | Duration of intervention | Participants characteristics | | | | | | | Mean (SD) at post-intervention | |
| --- | --- | --- | --- | --- | --- | --- | --- | --- | --- | --- | --- | --- | --- |
|  |  |  |  |  | Health condition | Digital solution group | | | Control/no digital solution group | | | Digital solution group | Control/no digital solution group |
|  |  |  |  |  |  | Age, mean (SD) | n | Females, n (%) | Age, mean (SD) | n | Females, n (%) |  |  |
| Boyne et al (2014) [15] | 12-item European Heart Failure Self Care Behavior Scale (EHFSCB). | Remote monitoring | Usual Care | Not clear (considerei 3 months) | Heart failure | 71.0 (11.9) | 197 | 82 (41.6) | 71.9 (10.5) | 185 | 74 (40) | 17.4 (6.1) | 20.0 (5.1) |
| Jung and Lee (2017) [9] | 10-item self-report instrument to assess key self-care behaviours | Remote monitoring + Website+face-to-face | Education | 24 weeks | Living alone with hypertension | 80.9 (6.6) | 31 | 24 (77.4) | 81.2 (4.1) | 33 | 29 (87.9) | 38.5 (4.1) | 34.9 (4.7) |
| Simbrig et al (2022) [25] | Assistive Technologies Quality of Life (ATQoL) Scale (participation item) | Remote monitoring+ Mobile app – Specific+ Website | No intervention | 6 and 13 months | No specific condition | 76.5 (8.1) | 143 | 95 (66.4) | 77.9 (7.6) | 138 | 99 (71.7) | No data provided | No data provided |
| Boer et al (2019) [20] | Telephonic Exacerbation Assessment System (TEXAS) | Remote monitoring + Mobile app - Specific | Usual Care | 12 months | No specific conditions | 69.3 (8.8) | 43 | 18 (41.9) | 65.9 (8.9) | 44 | 15 (34.1) | Contact health care professional n=61 (32.6%) Start prednisolone and/or antibiotics n=64 (34.2%)  Increase bronchodilator use n=135 (72.2%) | Contact health care professional n=68 (35.8%) Start prednisolone and/or antibiotics n=62 (32.6%)  Increase bronchodilator use n=135 (71.1%) |

Legend: SD – Standard Deviation; n – sample

## References

1. T. Bieg, C. Gerdenitsch, I. Schwaninger, B. M. J. Kern, and C. Frauenberger, “Evaluating Active and Assisted Living technologies: Critical methodological reflections based on a longitudinal randomized controlled trial,” *Comput Human Behav*, vol. 133, p. 107249, Aug. 2022, doi: 10.1016/j.chb.2022.107249.
2. D. H. Gustafson *et al.*, “Effect of an eHealth intervention on older adults’ quality of life and health-related outcomes: a randomized clinical trial,” *J Gen Intern Med*, vol. 37, no. 3, pp. 521–530, Feb. 2022, doi: 10.1007/s11606-021-06888-1.
3. K. H. Lee, Y. Y. Choi, and E. S. Jung, “Effectiveness of an oral health education programme using a mobile application for older adults: A randomised clinical trial,” *Gerodontology*, vol. 40, no. 1, pp. 47–55, Mar. 2023, doi: 10.1111/ger.12616.
4. T. Timmers *et al.*, “Assessing the Efficacy of an Educational Smartphone or Tablet App With Subdivided and Interactive Content to Increase Patients’ Medical Knowledge: Randomized Controlled Trial,” *JMIR Mhealth Uhealth*, vol. 6, no. 12, p. e10742, Dec. 2018, doi: 10.2196/10742.
5. Y. Guo, Y. Chen, D. A. Lane, L. Liu, Y. Wang, and G. Y. H. Lip, “Mobile Health Technology for Atrial Fibrillation Management Integrating Decision Support, Education, and Patient Involvement: mAF App Trial,” *Am J Med*, vol. 130, no. 12, pp. 1388-1396.e6, Dec. 2017, doi: 10.1016/j.amjmed.2017.07.003.
6. L. L. Y. Tsai, R. J. McNamara, C. Moddel, J. A. Alison, D. K. McKenzie, and Z. J. McKeough, “Home‐based telerehabilitation via real‐time videoconferencing improves endurance exercise capacity in patients with COPD: The randomized controlled TeleR Study,” *Respirology*, vol. 22, no. 4, pp. 699–707, May 2017, doi: 10.1111/resp.12966.
7. Y. Jiang *et al.*, “Evaluating an Intervention Program Using WeChat for Patients With Chronic Obstructive Pulmonary Disease: Randomized Controlled Trial,” *J Med Internet Res*, vol. 22, no. 4, p. e17089, Apr. 2020, doi: 10.2196/17089.
8. A. K. C. Wong, F. K. Y. Wong, K. K. S. Chow, S. M. Wong, and P. H. Lee, “Effect of a Telecare Case Management Program for Older Adults Who Are Homebound During the COVID-19 Pandemic,” *JAMA Netw Open*, vol. 4, no. 9, p. e2123453, Sep. 2021, doi: 10.1001/jamanetworkopen.2021.23453.
9. H. Jung and J.-E. Lee, “The impact of community-based eHealth self-management intervention among elderly living alone with hypertension,” *J Telemed Telecare*, vol. 23, no. 1, pp. 167–173, Jan. 2017, doi: 10.1177/1357633X15621467.
10. C. Knaevelsrud, M. Böttche, R. H. Pietrzak, H. J. Freyberger, and P. Kuwert, “Efficacy and Feasibility of a Therapist-Guided Internet-Based Intervention for Older Persons with Childhood Traumatization: A Randomized Controlled Trial,” *The American Journal of Geriatric Psychiatry*, vol. 25, no. 8, pp. 878–888, Aug. 2017, doi: 10.1016/j.jagp.2017.02.024.
11. B. J. Wakefield *et al.*, “Outcomes of a Home Telehealth Intervention for Patients with Diabetes and Hypertension,” *Telemedicine and e-Health*, vol. 18, no. 8, pp. 575–579, Oct. 2012, doi: 10.1089/tmj.2011.0237.
12. J. A. Sanford, P. C. Griffiths, P. Richardson, K. Hargraves, T. Butterfield, and H. Hoenig, “The Effects of In‐Home Rehabilitation on Task Self‐Efficacy in Mobility‐Impaired Adults: A Randomized Clinical Trial,” *J Am Geriatr Soc*, vol. 54, no. 11, pp. 1641–1648, Nov. 2006, doi: 10.1111/j.1532-5415.2006.00913.x.
13. C. Emme *et al.*, “The impact of virtual admission on self‐efficacy in patients with chronic obstructive pulmonary disease – a randomised clinical trial,” *J Clin Nurs*, vol. 23, no. 21–22, pp. 3124–3137, Nov. 2014, doi: 10.1111/jocn.12553.
14. Li *et al.*, “A WeChat-Based Self-Management Intervention for Community Middle-Aged and Elderly Adults with Hypertension in Guangzhou, China: A Cluster-Randomized Controlled Trial,” *Int J Environ Res Public Health*, vol. 16, no. 21, p. 4058, Oct. 2019, doi: 10.3390/ijerph16214058.
15. J. J. Boyne, H. J. Vrijhoef, M. Spreeuwenberg, G. De Weerd, J. Kragten, and A. P. Gorgels, “Effects of tailored telemonitoring on heart failure patients’ knowledge, self-care, self-efficacy and adherence: A randomized controlled trial,” *European Journal of Cardiovascular Nursing*, vol. 13, no. 3, pp. 243–252, Jun. 2014, doi: 10.1177/1474515113487464.
16. T. W. Kenealy *et al.*, “Telecare for Diabetes, CHF or COPD: Effect on Quality of Life, Hospital Use and Costs. A Randomised Controlled Trial and Qualitative Evaluation,” *PLoS One*, vol. 10, no. 3, p. e0116188, Mar. 2015, doi: 10.1371/journal.pone.0116188.
17. Z. D. Gellis, B. L. Kenaley, and T. Ten Have, “Integrated Telehealth Care for Chronic Illness and Depression in Geriatric Home Care Patients: The Integrated Telehealth Education and Activation of Mood (I‐TEAM) Study,” *J Am Geriatr Soc*, vol. 62, no. 5, pp. 889–895, May 2014, doi: 10.1111/jgs.12776.
18. S. Barnason, L. Zimmerman, J. Nieveen, M. Schmaderer, B. Carranza, and S. Reilly, “Impact of a home communication intervention for coronary artery bypass graft patients with ischemic heart failure on self-efficacy, coronary disease risk factor modification, and functioning,” *Heart & Lung*, vol. 32, no. 3, pp. 147–158, May 2003, doi: 10.1016/S0147-9563(03)00036-0.
19. E.-S. Nahm *et al.*, “The Effects of a Theory-Based Patient Portal e-Learning Program for Older Adults with Chronic Illnesses,” *Telemedicine and e-Health*, vol. 25, no. 10, pp. 940–951, Oct. 2019, doi: 10.1089/tmj.2018.0184.
20. L. Boer *et al.*, “A Smart Mobile Health Tool Versus a Paper Action Plan to Support Self-Management of Chronic Obstructive Pulmonary Disease Exacerbations: Randomized Controlled Trial,” *JMIR Mhealth Uhealth*, vol. 7, no. 10, p. e14408, Oct. 2019, doi: 10.2196/14408.
21. J. Fanning *et al.*, “The Effects of a Pain Management-Focused Mobile Health Behavior Intervention on Older Adults’ Self-efficacy, Satisfaction with Functioning, and Quality of Life: a Randomized Pilot Trial,” *Int J Behav Med*, vol. 29, no. 2, pp. 240–246, Apr. 2022, doi: 10.1007/s12529-021-10003-3.
22. D. Bossen, C. Veenhof, K. E. Van Beek, P. M. Spreeuwenberg, J. Dekker, and D. H. De Bakker, “Effectiveness of a Web-Based Physical Activity Intervention in Patients With Knee and/or Hip Osteoarthritis: Randomized Controlled Trial,” *J Med Internet Res*, vol. 15, no. 11, p. e257, Nov. 2013, doi: 10.2196/jmir.2662.
23. P. Bernocchi *et al.*, “Feasibility and Clinical Efficacy of a Multidisciplinary Home-Telehealth Program to Prevent Falls in Older Adults: A Randomized Controlled Trial,” *J Am Med Dir Assoc*, vol. 20, no. 3, pp. 340–346, Mar. 2019, doi: 10.1016/j.jamda.2018.09.003.
24. R. L. H. Berman, M. A. Iris, R. Bode, and C. Drengenberg, “The Effectiveness of an Online Mind-Body Intervention for Older Adults With Chronic Pain,” *J Pain*, vol. 10, no. 1, pp. 68–79, Jan. 2009, doi: 10.1016/j.jpain.2008.07.006.
25. I. M. Simbrig, S. van der Weegen, F. Piazolo, M. Kofler, N. Sturm, and S. Hvalič-Touzery, “Impact of using aging-in-place technologies on quality of life: Results from a randomized controlled trial in four European countries,” *Gerontechnology*, vol. 21, no. 1, pp. 1–21, Jan. 2022, doi: 10.4017/gt.2023.21.1.792.02.
26. A. K. C. Wong, J. Bayuo, F. K. Y. Wong, K. K. S. Chow, S. M. Wong, and A. C. K. Lau, “The Synergistic Effect of Nurse Proactive Phone Calls With an mHealth App Program on Sustaining App Usage: 3-Arm Randomized Controlled Trial,” *J Med Internet Res*, vol. 25, p. e43678, May 2023, doi: 10.2196/43678.
27. Z. Yuting, T. Xiaodong, and W. Qun, “Effectiveness of a mHealth intervention on hypertension control in a low-resource rural setting: A randomized clinical trial,” *Front Public Health*, vol. 11, Mar. 2023, doi: 10.3389/fpubh.2023.1049396.
28. M. L. Lim *et al.*, “A Self-Guided Online Cognitive Behavioural Therapy to Reduce Fear of Falling in Older People: a Randomised Controlled Trial,” *Int J Behav Med*, vol. 30, no. 3, pp. 455–462, Jun. 2023, doi: 10.1007/s12529-022-10105-6.
29. A. K. C. Wong, F. K. Y. Wong, K. K. S. Chow, S. M. Wong, J. Bayuo, and A. K. Y. Ho, “Effect of a Mobile Health Application With Nurse Support on Quality of Life Among Community-Dwelling Older Adults in Hong Kong,” *JAMA Netw Open*, vol. 5, no. 11, p. e2241137, Nov. 2022, doi: 10.1001/jamanetworkopen.2022.41137.
30. M. F. Muldoon *et al.*, “Randomized feasibility trial of a digital intervention for hypertension self-management,” *J Hum Hypertens*, vol. 36, no. 8, pp. 718–725, Aug. 2022, doi: 10.1038/s41371-021-00574-9.
